# Deciphering the Microbiota of Edible Insects Sold by Street Vendors in Thailand Using Metataxonomic Analysis

**DOI:** 10.3390/insects16020122

**Published:** 2025-01-26

**Authors:** Giorgia Rampanti, Federica Cardinali, Ilario Ferrocino, Vesna Milanović, Cristiana Garofalo, Andrea Osimani, Lucia Aquilanti

**Affiliations:** 1 Dipartimento di Scienze Agrarie, Alimentari ed Ambientali, Università Politecnica delle Marche, Via Brecce Bianche, 60131 Ancona, Italy; g.rampanti@univpm.it (G.R.); f.cardinali@univpm.it (F.C.); v.milanovic@univpm.it (V.M.); c.garofalo@univpm.it (C.G.); l.aquilanti@univpm.it (L.A.); 2 Department of Agricultural, Forest, and Food Science, University of Turin, Largo Paolo Braccini 2, Grugliasco, 10095 Torino, Italy; ilario.ferrocino@unito.it

**Keywords:** microbial ecology, ethnic foods, ready-to-eat insects, traditional foods, fried insects

## Abstract

The aim of the present study was to investigate the microbiota of processed ready-to-eat (fried or boiled) edible insects sold by street vendors at local green markets in Thailand (Bangkok and Koh Samui). To this end, samples of 4 insect species (rhino beetle adults, silkworm pupae, giant waterbugs adults, and black scorpions) were collected and analyzed through viable counting and metataxonomic analysis. Enterobacteriaceae showed counts below 1 log cfu g^−1^ in all samples, except for black scorpions, which showed elevated counts reaching up to 4 log cfu g^−1^. Total mesophilic aerobes counts were up to 8 log cfu g^−1^ in all the analyzed samples. Counts below 1 log cfu g^−1^ were observed for *Escherichia coli*, *Staphylococcus aureus*, sulfite-reducing clostridia viable cells and spores, and *Bacillus cereus*. All the samples showed the absence of *Listeria monocytogenes* and *Salmonella* spp. According to metataxonomic analysis, 14 taxa were consistently present across all insect samples, including *Dellaglioa algida*, *Latilactobacillus curvatus*, *Latilactobacillus sakei*, Acetobacteraceae, *Apilactobacillus kunkeei*, *Bombilactobacillus* spp., Enterobacteriaceae, *Gilliamella* spp., *Lactobacillus* spp., *Lactobacillus apis*, *Streptococcus thermophilus*, *Lacticaseibacillus rhamnosus*, *Lactiplantibacillus plantarum*, and *Weissella* spp. Minority taxa included *Alcaligenes* spp., *Brochothrix thermosphacta*, *Psychrobacter* spp., *Staphylococcus saprophyticus*, *Lactobacillus melliventris*, *Pediococcus* spp., *Levilactobacillus brevis*, and *Snodgrassella alvi*.

## Simple Summary

Edible insects have recently garnered significant interest from the market and researchers due to their potential as a sustainable source of proteins, amino acids, and minerals. However, one of the primary concerns associated with edible insects is their microbial contamination. In Thailand, edible insects are commonly sold at green markets, attracting both residents and tourists. To the authors’ knowledge, no studies have been conducted on the microbiota of edible insects sold by street vendors in Thailand. This study investigates the microbiota of processed (fried or boiled), ready-to-eat edible insects sold by street vendors at local markets in Bangkok and Koh Samui, Thailand, using viable counting and metataxonomic analysis. On a positive note, the culture-dependent analyses revealed no safety concerns, showing the absence of major foodborne pathogens such as *Listeria monocytogenes* and *Salmonella* spp., as well as spore-forming bacteria. Notably, except for black scorpions, which were sold after boiling, hygiene indicator microorganisms like Enterobacteriaceae were below detection limits in all fried samples. This suggests that frying was more effective than boiling in reducing the microbial load. Metataxonomic analysis further confirmed the absence of pathogenic taxa and unexpectedly revealed that lactic acid bacteria constituted the predominant fraction of the microbiota.

## 1. Introduction

Edible insects have recently garnered significant attention from both the market and the scientific community in Europe. Recognized as a sustainable source of proteins, amino acids, and minerals, they have been recently included in the European Union’s list of authorized novel foods [1]. Conversely, edible insects have long been used as food source in Latin American, African, and Southeast Asian countries, where they are considered ethnic foods [1]. Edible insects can be consumed whole (either fresh or processed through drying, roasting, boiling, or frying), in the form of powders or pastes, or they can be used for the extraction of proteins, lipids, or fibers.

Among the 2000 species of surveyed edible insects, from 150 to 200 are shared among Indonesia, Lao PDR, Malaysia, Myanmar, the Philippines, and Vietnam, whereas Thailand accounts for 194 species, with a total of 81 insects currently eaten in this country [2]. In Thailand, edible insects ready for consumption are available in both large shopping centers and small local markets. These points of sale capture the interest of Western tourists, who are intrigued by products still often viewed with disgust and skepticism in their home countries [3], as well as by local consumers seeking a nostalgic connection [1]. However, beyond the folkloristic aspect, ready-to-eat edible insects available in shopping centers or sold by street vendors raise serious questions about their safety.

The primary risks associated with edible insects for consumers stem from chemical contaminants, such as pesticides, toxins, and heavy metals, which can lead to irritation, acute or chronic intoxication, and potentially mutagenic or carcinogenic effects. Additionally, biological contaminants pose a concern, including mycotoxins, bacterial toxins, and microbial species found on the exoskeleton or in the gut. These microbes may be symbiotic, commensal, pathogenic, or potentially pathogenic [4]. As reported by Garofalo et al. [4] and Osimani & Aquilanti [5], the microbiota of raw edible insects is very complex, being mainly characterized by Enterobacteriaceae (e.g., *Enterobacter*, *Erwinia*, *Klebsiella*, *Pantoea*, etc.), lactic acid bacteria (e.g., *Enterococcus*, *Lactobacillus*, *Lactococcus*, *Pediococcus*, *Weissella*, etc.), spore-forming bacteria (e.g., *Bacillus* and *Clostridium*), pseudomonads, and staphylococci. However, the occurrence of different taxa is strongly influenced by many factors, including the rearing conditions, the feed source, the insect species, and the processing treatment applied to the insects [6,7].

The microbiota of foods can be studied using conventional culture-dependent methods, such as viable counting on selective or elective culture media. However, many microbial species lack suitable culture media, resulting in incomplete or partial findings. Regarding edible insects, numerous studies conducted between 2000 and 2016 primarily focused on viable counting and reported the presence of eumycetes and total mesophilic aerobes [8,9,10,11,12,13,14,15]. A few of these studies also isolated certain cultivable taxa, which were occasionally characterized using physiological methods. However, these studies have provided only a partial overview of the complex microbial communities present in edible insects. Conversely, culture-independent techniques, based on the study of microbial DNA, can provide a more complete overview of microbial populations in a given sample. Among these latter methods, metataxonomic analysis represents the most advanced method to obtain a complete overview of microbial populations occurring in complex food matrices [16]. Regarding edible insects, the use of metataxonomic analysis, applied to this novel food source primarily in Europe since 2016, has led to the discovery of both major and minor microbial taxa [17,18,19,20,21,22]. This has enabled the research community and the food industry to more effectively assess microbiological risks associated with this emerging food source [23,24,25].

Although the microbiota of edible insects sold in the European Union has widely been investigated, to the authors’ knowledge a lack of investigation on the microbiota of edible insects sold by street vendors located in Thailand is observed. Hence, the aim of the present study was to investigate the microbiota of processed ready-to-eat edible insects sold by street vendors at local markets in Thailand (Bangkok and Koh Samui). To this end, samples of 4 insect species were collected and analyzed through viable counting and metataxonomic analysis. Although performed with different samples and different collection sites, this research serves as a continuation of the study on the same Thai insect species originally initiated by Milanović et al. [26].

## 2. Materials and Methods

### 2.1. Collection of Samples

Four types of ready-to-eat Thai insects were collected for this study as follows: A—rhino beetle (*Hyboschema contractum*) adults collected in Koh Samui, B—silkworm (*Bombyx mori*) pupae collected in Bangkok, C—silkworm (*B. mori*) pupae collected in Koh Samui, D—giant waterbugs (*Belostomatidae*) adults collected in Koh Samui, and E—black scorpions (*Heterometrus longimanus*) collected in Bangkok. For each type of insect (A–E), two samples were collected.

All insect samples were purchased from street vendors at local green markets in the year 2022. The insects were deep-fried in cooking oil at ~180 °C, except for black scorpions that were provided boiled and dried. All samples were slightly salted. The street vendors did not provide any information about the origin of the insects, whether they were wild-collected or farmed. As already performed by Köhler et al. [1], the insects were placed in sealed food-grade plastic bags, then frozen and packed in a Styrofoam box with ice packs for preservation. The samples were then shipped to Italy though international express courier and stored under frozen conditions (−20 °C) until analyses.

The unused samples that were not analyzed were disposed of in a controlled manner as special laboratory biological waste.

### 2.2. Water Activity

The water activity (a_w_) of the analyzed insect samples was measured according to the ISO 21807:2004 method using an AwTherm apparatus (Rotronic, Bassersdorf, Switzerland).

### 2.3. Microbial Counts

Viable counts were carried out by adding 10 g of each sample to 90 mL of sterile peptone water (1 g L^−1^ of bacteriological peptone); homogenization was performed with a Stomacher apparatus (400 Circulator, International PBI, Milan, Italy) for 3 min at 260 rpm. Then, ten-fold serial dilutions were prepared, and viable counts of the following microorganisms were evaluated as follows: (i) Enterobacteriaceae in Violet Red Bile Glucose Agar (VRBGA) (VWR, Leuven, Belgium) incubated for 24 h at 37 °C; (ii) *Escherichia coli* in Chromogenic Coliform Agar (CCA) medium (VWR) incubated for 24 h at 37 °C; (iii) *Staphylococcus aureus* in CHROMagar™ *Staphylococcus* (VWR) incubated for 24 h at 37 °C; (iv) total mesophilic aerobes in Plate Count Agar (PCA) (VWR) incubated for 48–72 h at 30 °C; (v) *Bacillus cereus* in CHROMagar™ *B. cereus* (VWR) incubated for 24 h at 30 °C.

Sulfite-reducing clostridia were counted in Tryptone Sulfite Neomycin (TSN) agar (Liofilchem, Teramo, Italy) and incubated for 24 h at 37 °C under anaerobic conditions using the AnaeroGen 2.5 System. Moreover, regarding sulfite-reducing clostridia spores, homogenates were previously treated in a water bath at 80 °C for 10 min and cooled in ice; aliquots of the serial ten-fold dilutions of the processed samples were inoculated in TSN agar (Liofilchem) and incubated at for 24 h at 37 °C under anaerobiosis.

The results of two biological replicates were expressed as the log of colony forming units (cfu) per gram of sample and reported as mean ± standard deviation.

A miniVIDAS (bioMérieux, Marcy-l’Étoile, France) apparatus was used to assess the presence or absence of *Listeria monocytogenes* and *Salmonella* spp. using the Enzyme-Linked Fluorescent Assay (ELFA), according to the standard AFNOR BIO 12/11–03/04 and AFNOR BIO 12/16–09/05 methods [27].

### 2.4. DNA Extraction, Amplificon-Based Sequencing, and Metataxonomic Analysis

The microbial DNA was extracted directly from the edible insect samples using a PowerFood™ Microbial DNA Isolation Kit (Mo Bio Laboratories, Carlsbad, CA, USA). In detail, 1 mL of each insect homogenate (dilution 10^−1^) was centrifuged to produce a pellet that was processed according to the kit manufacturer’s instructions. The quality of the extracted DNA was verified using a NanoDrop spectrophotometer (Thermo Scientific, Milan, Italy).

DNA directly extracted from insect samples was used to assess the microbiota by amplifying the V3-V4 region of the *16S rRNA* gene using primers and protocols as outlined by Klindworth et al. [28].

The PCR amplicons were then purified with an Agencourt AMPure kit (Beckman Coulter, Milan, Italy), and sequencing adapters were added to the clean PCR products using the Nextera XT Library Preparation Kit (Illumina Inc., San Diego, CA, USA), following the manufacturer’s protocols.

Following the second purification step, PCR products were quantified using a QUBIT dsDNA Assay kit (Life Technologies, Milan, Italy). Subsequently, amplicons were pooled at equimolar concentrations (4 nM). The library was denatured using 0.2 N NaOH, then diluted to a concentration of 12 pM and mixed with 20% (*v*/*v*) denatured 12 pM PhiX, following the preparation instructions provided by Illumina.

Sequencing was conducted on a MiSeq instrument (Illumina) with V3 chemistry, producing 2 × 250 bp paired-end reads. MiSeq Control Software v2.3.0.3, RTA v1.18.42.0, and CASAVA v1.8.2 were utilized for base calling and Illumina barcode demultiplexing.

Paired-end reads were imported in QIIME2 software version 2024.10 [29]. Briefly, primers were removed by Cutadapter and sequences were trimmed for low quality, filtered from chimeric sequences, and merged by using the dada2 denoise-paired plug in of QIIME2 [30]. Taxonomy classification was performed against the Greengenes *16S rRNA* gene database by means of the QIIME2 feature-classifier. Amplicon sequence variants (ASVs) with less than five read counts in at least two samples were excluded to increase the confidence of sequence reads.

The raw reads data were deposited in the Sequence Read Archive of NCBI under the BioProject accession number PRJNA1185947.

### 2.5. Statistical Analysis

All microbiological data were log-transformed prior to statistical analysis. For samples with no colonies on agar plates, the approach outlined by Jones and Anderson [31] was followed, assigning a zero value to plate counts showing no growth after log transformation [32].

The results of a_w_ and microbial counts were analyzed using one-way analysis of variance (ANOVA), with significant differences between samples identified using Tukey-Kramer’s Honest Significant Difference (HSD) post-hoc test (*p* < 0.05). Statistical analyses were performed using JMP software (version 11.0.0; SAS Institute Inc., Cary, NC, USA).

Alpha and Beta diversity indices of bacterial populations were calculated through the diversity script of QIIME2 using rarefied counts. Differences between alpha diversity parameter and ASVs frequency were analyzed by non-parametric Kruskall-Wallis test in R environment. Differences in Bray-Curtis index were evaluated by pairwise PERMANOVA. Venn diagram was obtained with the VennDiagram package in R environment.

## 3. Results

### 3.1. Water Activity

The results of a_w_ measurements are reported in Table 1.

In more detail, the values ranged between 0.91 ± 0.01 and 0.97 ± 0.00 with significant higher average value for rhino beetles.

### 3.2. Microbial Counts

The results of viable counts are reported in Table 1.

For Enterobacteriaceae, counts below the detection limit of 1 log cfu g^−1^ were observed in all samples, with the exception of black scorpion samples, which showed elevated counts reaching up to 4 log cfu g^−1^.

Regarding total mesophilic aerobes, counts up to 8 log cfu g^−1^ were observed in all the analyzed samples, with no statistically significant differences among samples (*p* < 0.05).

Counts below the detection limit of 1 log cfu g^−1^ were observed for *E. coli*, *S. aureus*, sulfite-reducing clostridia viable cells and spores, and *B. cereus*.

*L. monocytogenes* and *Salmonella* spp. were not detected in any of the samples.

### 3.3. Taxonomic Diversity

**Figure 1:**
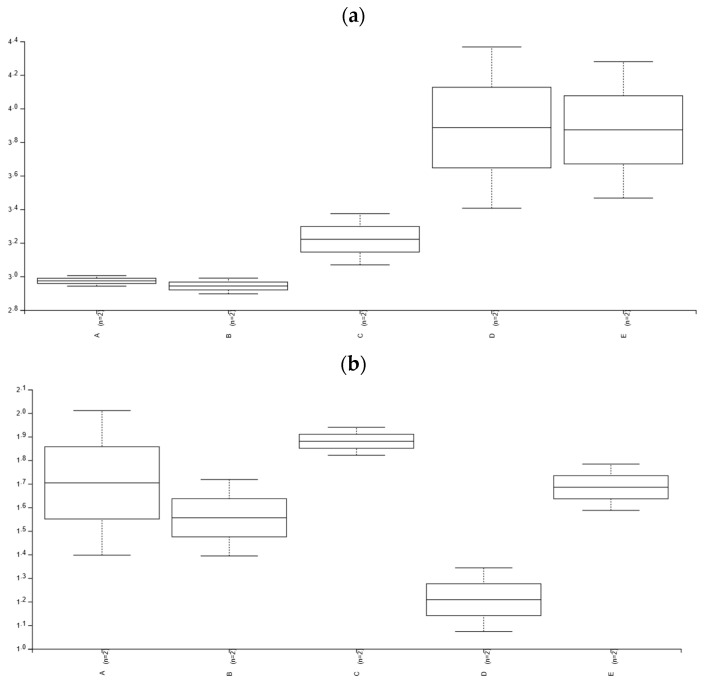

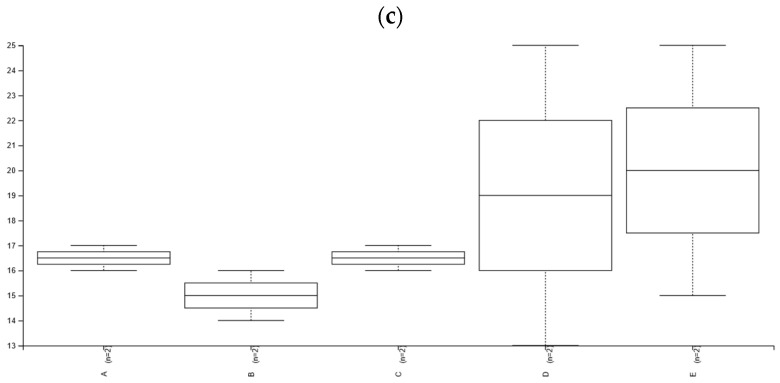
Alpha diversity plots showing the Shannon diversity index (**a**), Faith’s PD index (**b**), and Observed ASV index (**c**) of the bacterial community in different samples (A, B, C, D, E). A—rhino beetle (*Hyboschema contractum*) adults collected in Koh Samui. B—silkworm (*Bombyx mori*) pupae collected in Bangkok. C—silkworm (*B. mori*) pupae collected in Koh Samui. D—giant waterbugs (*Belostomatidae*) adults collected in Koh Samui. E—black scorpions (*Heterometrus longimanus*) collected in Bangkok.

A total of 144,331 reads were analyzed, with an average of 14,433 reads per sample. No statistically significant differences in alpha diversity, as measured by the Shannon index (Figure 1a), Faith’s PD index (Figure 1b), and Observed ASV index (Figure 1c) were observed among the samples (*p* > 0.05).

**Figure 2:**
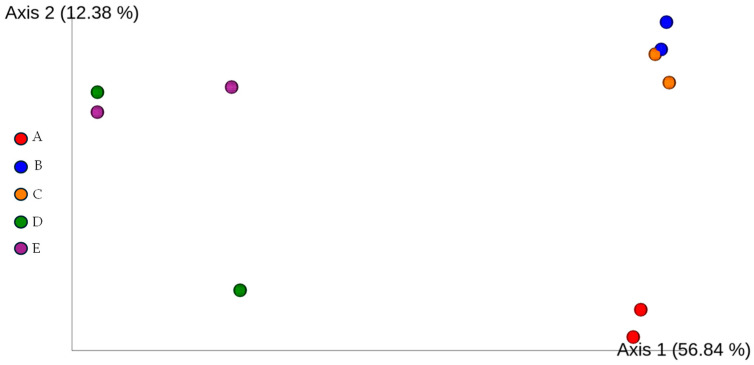
Bray-Curtis dissimilarity-based Emperor ordination plot illustrating the beta diversity of microbial communities in different samples (A, B, C, D, E). A—rhino beetle (*Hyboschema contractum*) adults collected in Koh Samui. B—silkworm (*Bombyx mori*) pupae collected in Bangkok. C—silkworm (*B. mori*) pupae collected in Koh Samui. D—giant waterbugs (*Belostomatidae*) adults collected in Koh Samui. E—black scorpions (*Heterometrus longimanus*) collected in Bangkok.

No significant differences were observed in the Bray-Curtis dissimilarity index among groups (*p* > 0.05). The ordination plots are illustrated in Figure 2.

**Figure 3:**
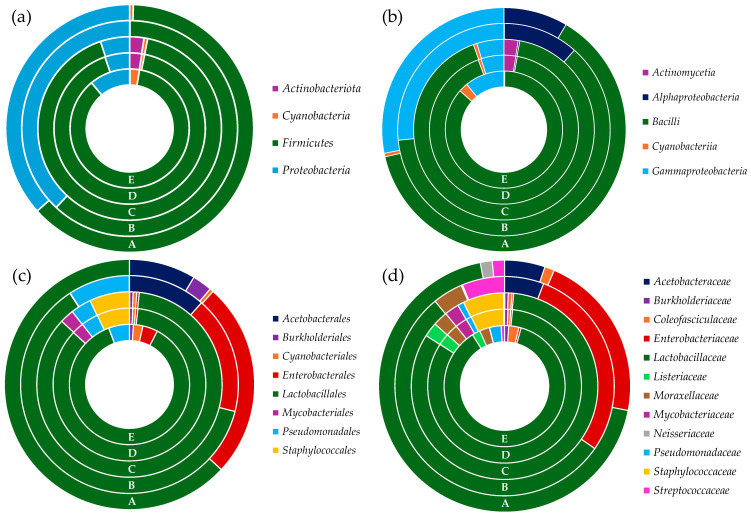
Microbial composition of edible insect samples at different taxonomic levels. (**a**) Phylum level; (**b**) Class level; (**c**) Order level; (**d**) Family level. A—rhino beetle (*Hyboschema contractum*) adults collected in Koh Samui. B—silkworm (*Bombyx mori*) pupae collected in Bangkok. C—silkworm (*B. mori*) pupae collected in Koh Samui. D—giant waterbugs (*Belostomatidae*) adults collected in Koh Samui. E—black scorpions (*Heterometrus longimanus*) collected in Bangkok.

Figure 3 shows the distribution of microbial communities at different taxonomic levels across the five edible insect groups (A, B, C, D, E). At the phylum level (Figure 3a), Firmicutes dominated all groups (ranging from 62% to 92% of the relative frequencies), followed by Proteobacteria (ranging from 5% to 38% of the relative frequencies). Actinobacteriota and Cyanobacteria were detected in low abundance (<3%). At the class level (Figure 3b), the taxon Bacilli was dominant in all groups, followed by Gammaproteobacteria. Silkworm samples (B, C) were characterized by the presence of Actinomycetia (approximately 3% of the relative frequencies), whereas giant waterbugs (D) and black scorpions (E) showed the presence of Alphaproteobacteria, accounting for 11% and 8% of the relative frequencies, respectively. At the order level (Figure 3c), the taxon Lactobacillales emerged as the dominant taxon in all insect groups. In rhino beetle samples (A), this taxon was followed by Pseudomonadales, Enterobacterales, Cyanobacteriales, and Burkholderiales. In silkworm samples (B, C), Lactobacillales were followed by Staphylococcales, Pseudomonadales, Mycobacteriales, Burkholderiales, Cyanobacteriales, and Enterobacterales. In giant waterbugs and black scorpion samples (D, E), the order Lactobacillales was followed by Enterobacterales (17% and 25% of the relative frequencies, respectively) and Acetobacterales (11% and 9% of the relative frequencies, respectively). Giant waterbugs (D) also showed the presence of Pseudomonadales (9%), whereas black scorpions (E) were characterized by the presence of Burkholderiales and Cyanobacteriales. The microbial distribution at the family level (Figure 3d) showed that Lactobacillaceae were dominant in all groups. In rhino beetle samples (A) this taxon was followed by Coleofasciculaceae, Moraxellaceae, Pseudomonadaceae, Listeriaceae, Burkholderiaceae, Streptococcaceae, and Enterobacteriaceae. In silkworm samples (B, C), Lactobacillaceae were followed by Staphylococcaceae, Moraxellaceae, Listeriaceae, Mycobacteriaceae, Pseudomonadaceae, Coleofasciculaceae, Burkholderiaceae, and Enterobacteriaceae. Giant waterbugs and black scorpion samples (D, E) showed the common presence of Enterobacteriaceae, Acetobacteriaceae, and Streptococcaceae. Giant waterbugs (D) also showed the presence of Moraxellaceae, whereas black scorpions (E) were characterized by the presence of Neisseriaceae and Coleofasciculaceae.

**Figure 4:**
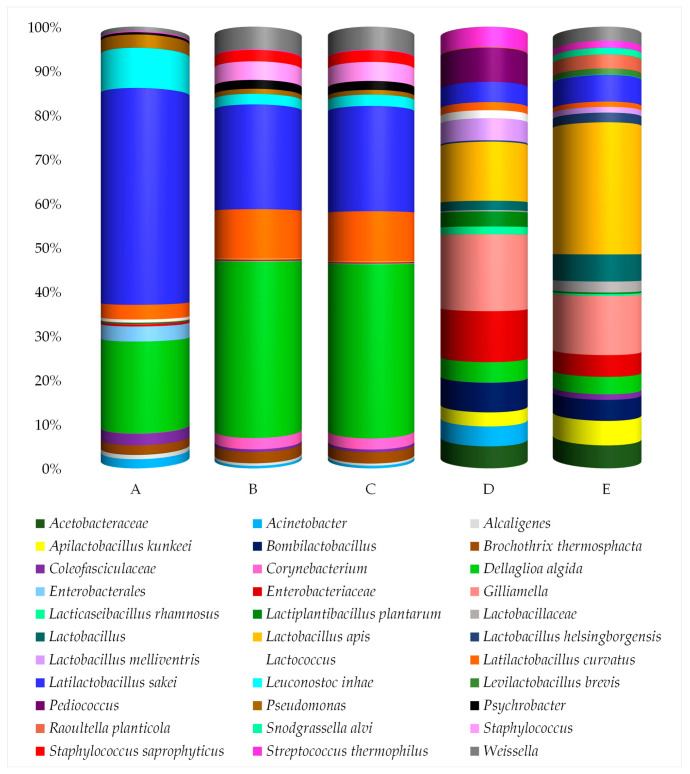
Relative abundance (%) of the taxa detected through *16S rRNA* sequencing. Only ASVs with a relative abundance of at least 0.2% in a minimum of two samples are shown. A—rhino beetle (*Hyboschema contractum*) adults collected in Koh Samui; B—silkworm (*Bombyx mori*) pupae collected in Bangkok; C—silkworm (*B. mori*) pupae collected in Koh Samui; D—giant waterbugs (*Belostomatidae*) adults collected in Koh Samui; E—black scorpions (*Heterometrus longimanus*) collected in Bangkok.

ASVs at the species level with a relative abundance of at least 0.2% in at least two samples are presented in Figure 4 and Table S1. When species-level resolution could not be achieved, the results are reported at higher taxonomic levels.

**Figure 5:**
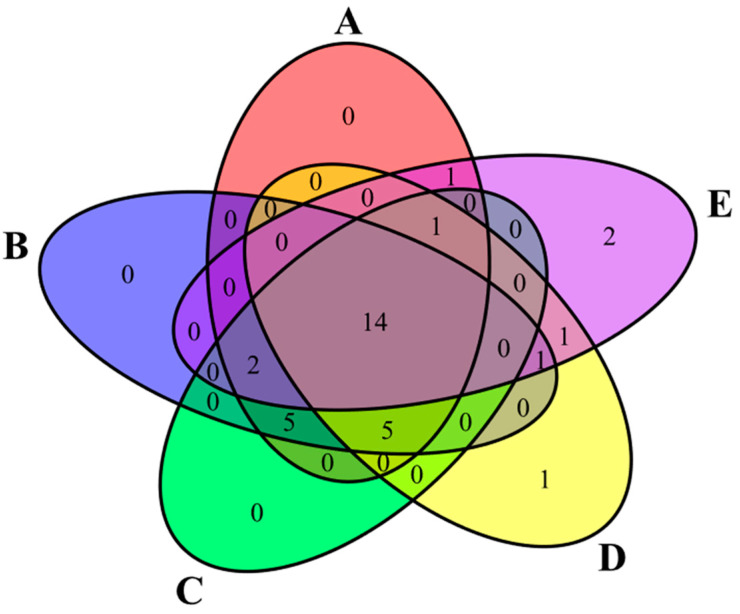
Venn diagram illustrating the overlap of bacterial ASVs among samples. In the diagram, the number of shared taxa is reported. A—rhino beetle (*Hyboschema contractum*) adults collected in Koh Samui. B—silkworm (*Bombyx mori*) pupae collected in Bangkok. C—silkworm (*B. mori*) pupae collected in Koh Samui. D—giant waterbugs (*Belostomatidae*) adults collected in Koh Samui. E—black scorpions (*Heterometrus longimanus*) collected in Bangkok.

As shown in Figure 5, 14 taxa were consistently present across all insect samples. In detail, the species *Dellaglioa algida* (ranging from 3.82 to 39.40% of the relative frequency), *Latilactobacillus curvatus* (1.15–11.29%), and *Latilactobacillus sakei* (4.50–48.43%) were among the most abundant taxa in all samples. Additional taxa, including Acetobacteraceae, *Apilactobacillus kunkeei*, *Bombilactobacillus* spp., Enterobacteriaceae, *Gilliamella* spp., *Lactobacillus* spp., *Lactobacillus apis*, and *Streptococcus thermophilus*, were also present in all samples but showed higher relative frequencies in samples of giant waterbugs (D) and black scorpions (E) compared to the other samples. Minor taxa, such as *Lacticaseibacillus rhamnosus*, *Lactiplantibacillus plantarum*, and *Weissella* spp., were detected in all samples with relative abundances ≤5.2%. Samples of rhino beetles (A), silkworm pupae collected in Bangkok (B), and silkworm pupae collected in Koh Samui (C) showed the common presence of *Alcaligenes* spp. (0.50–0.94%), *Brochothrix thermosphacta* (2.06–2.48%), Enterobacterales (0.13–3.38%), *Psychrobacter* spp. (0.51–2.00%), and *Staphylococcus saprophyticus* (0.01–2.58%). In contrast, *Lactobacillus melliventris* was detected exclusively in samples of giant waterbugs (D) and black scorpions (E), with relative frequencies of 4.89 and 1.28%, respectively. Furthermore, samples of giant waterbugs (D) also showed the presence of *Pediococcus* spp., accounting for 7.57% of the relative frequency, whereas samples of black scorpions (E) showed the presence of *Levilactobacillus brevis* and *Snodgrassella alvi*, attesting at 1.35 and 1.49% of the relative frequency, respectively.

Other taxa, including *Acinetobacter* spp., Coleofasciculaceae, *Corynebacterium* spp., Lactobacillaceae, *Lactobacillus helsingborgensis*, *Lactococcus* spp., *Leuconostoc inhae*, *Pseudomonas* spp., *Raoultella planticola*, and *Staphylococcus* spp. were identified at low relative abundances (<10% in total).

## 4. Discussion

As recently reported by Wu et al. [33], the street food industry is expanding rapidly, creating employment chances, generating profits, and promoting tourism expansion. However, food prepared by street vendors can harbor microorganisms, and incidents of food poisoning associated with street food consumption have occasionally been reported [33].

In the present study, viable microbial counting and metataxonomic analysis of ready-to-eat processed edible insects from street vendors in local markets in Bangkok and Koh Samui, Thailand, revealed the presence of living microbial populations within these unique food matrices.

Of note, one of the main factors affecting the viability of microorganisms in foodstuffs is the presence of water. Water activity (a_w_) is often described as the measure of free or unbound water in a substance. In more detail, a_w_ of a food expresses the ratio between the vapor pressure of the food, when it is in complete undisturbed equilibrium with the surrounding air, and the vapor pressure of distilled water under the same exact conditions. Bacteria generally need water activity levels of at least 0.91 to grow, whereas fungi can grow at levels as low as 0.6. Each microorganism has a specific minimum water activity threshold below which growth is inhibited [34]. To lower a_w_, and ensure preservation of food from microbial growth, ingredients such as sodium chloride, sucrose, alcohol, propylene glycol, or glycerin can be added; moreover, thermal treatments can also be applied.

In the present study, all the a_w_ values detected in the analyzed samples were comprised in the range of those generally able to sustain microbial growth.

Frying and boiling of insects represent two out the most used thermal treatments to lower the microbial loads naturally occurring in this food [35]. Although a few papers dealing with the proximate composition of Thai edible insects have been published [1,36], to the author’s knowledge, a shortage of information regarding microbial loads of ready-to-eat edible insects sold by street vendors in Thailand is available in the scientific literature.

In the present study, regarding Enterobacteriaceae, that include *E. coli* and *Salmonella* spp., the levels (<1 log cfu g^−1^ or absence in 25 g for *Salmonella* spp.) detected in samples of rhino beetles (A), silkworm pupae (B and C), and giant waterbugs (D) attested the absence of enteric contamination. Enterobacteriaceae are a family of microorganisms that are often used as indicators of the application of good manufacturing practices (e.g., thermal treatment) [32]. Hence, based on the results, it is likely that the frying of insects was able to kill most of the microorganisms ascribed to Enterobacteriaceae. Regarding black scorpions (E), no *E. coli* or *Salmonella* spp. were detected, however, the high loads of Enterobacteriaceae detected in this insect deserve attention. Indeed, as reported by Brown et al. [37], the occurrence of such indicator microorganisms might indicate the presence of pathogens, as these latter may represent a subset of indicator organisms. It is noteworthy that the analyzed black scorpions were subjected to boiling instead of frying, hence, it is likely that in this case no effective thermal treatment (e.g., non-homogeneous heating of the insect) was applied. As reviewed by Garofalo et al. [4], the loads of Enterobacteriaceae harbored by unprocessed edible insects attest up to 7 log cfu g^−1^. However, the data obtained in the present study generally agreed with those reported by Klunder et al. [38] for fried and boiled edible insects, who found Enterobacteriaceae counts from 4 to 7 log cfu g^−1^ before processing and <1 log cfu g^−1^ after thermal treatment.

In all the samples, high counts of total mesophilic aerobes were observed. As reported by Petruzzelli et al. [32], this group of microorganisms is frequently used to evaluate the hygiene of the entire food production process. Hence, the high loads of viable mesophilic aerobes suggest improper handling or storage of the analyzed ready-to-eat insects after processing. The data herein reported are in agreement with those obtained by Klunder et al. [38] for fried and boiled insects, and with those observed by Milanović et al. [26] in fried edible insects industrially produced in Thailand, that showed total mesophilic aerobes counts from <1 to 7 log cfu g^−1^, depending on the insect species.

Although Osimani & Aquilanti [5] identified spore-forming bacteria as a common threat in edible insects, in the samples herein studied no concerning levels of these bacteria were evidenced.

Next-generation sequencing techniques are demonstrating significant utility in analyzing the microbiota of edible insects, a critical factor for ensuring food safety and harnessing beneficial insects-associated microorganisms [4]. For instance, a recent study on edible insects, including diving beetles, silkworms, grasshoppers, mealworms, and crickets, showed that these products may harbor both lactic acid bacteria and potential pathogens like *Klebsiella pneumoniae* and *Acinetobacter baumannii* [23]. Aleknavičius at al. [39] observed that the core microbiomes of crickets belonged to *Parabacteroides* and *Bacteroides* genera; *Lactococcus*, *Caprococcus*, *Enterococcus*, *Akkermansia*, and *Acinetobacter* were also found at low frequency [39]. The study by Frigerio et al. [40] on different insect-based food products made of house crickets, mealworm beetles, and lesser mealworms, revealed that Enterobacteriaceae, *Lactococcus*, *Enterobacter*, and *Enterococcus* were shared among all the samples. However, the authors found exclusive taxa based on the insect species.

In this study, the metataxonomic analysis identified a shared group of bacterial taxa, predominantly lactic acid bacteria, across the five insect species. It is important to note that these results may reflect DNA extracted from both living and dead cells, depending on the effectiveness of the thermal treatment (frying or boiling) or from cross-contamination occurring after processing.

As for *D. algida* (originally described as *Lactobacillus algidus*), to the authors’ knowledge, a lack of data regarding the presence of this microbial species in edible insects as well as in pest insects is observed. Of note, *L. algidus* has already been identified among the spoilage microorganisms in meat products; however, this species results still poorly characterized [41]. Interestingly, the metabolic activity of *L. algidus* in foodstuffs has been associated with the production of biogenic amines, including ornithine, putrescine, and tyramine [41], thus suggesting potential adverse effects on the consumers. In the insect samples herein analyzed, the occurrence of *D. algida* can be the result of cross-contamination by street vendors during the sale, or a lack of thermal treatment.

Regarding *L. curvatus*, this bacterial species has been found in fermented meat products, milk, grapes, and plant material [42]; however, to the author’s knowledge, no reports on the occurrence of *L. curvatus* in edible insects or pest insects are available in the scientific literature. Interestingly, Borremans et al. [43] have successfully used *L. curvatus* for Coleoptera (mealworms) paste fermentation, thus suggesting that the insect environment can represent a valid substrate for this species of lactic acid bacteria. The same discussion can be made considering the occurrence of *L. sakei*. Interestingly, *L. sakei* was recently isolated from honeybee (*Apis mellifera lamarckii* and *Apis mellifera carnica*) stomachs [44], thus suggesting a potential beneficial role of this species of lactic acid bacteria for the health of insects.

As for *A. kunkeei*, this species of lactic acid bacteria has shown to be well-adapted to the insect environment, having been previously isolated from honey, beebread, bee pollen, and honeybees as symbiont, with potential probiotic effect on the insect gut [45,46]. Therefore, a similar beneficial effect can be hypothesized for the insects herein studied.

Considering *Bombilactobacillus* spp., *Bombilactobacillus apium* has recently been isolated from the gut of honeybees (*Apis cerana*) [47] and has demonstrated a symbiotic activity in *Apis mellifera* [48]. Based on this evidence, a similar activity might be expected in the insects examined in this study.

The shared group of bacterial taxa detected in the analyzed insects also included *L. apis*. This species of lactic acid bacteria has already demonstrated to be well-adapted to the insect environment, having been isolated from the stomach of honeybees (*A. mellifera*) [49]. Intriguingly, *L. apis* demonstrated the capability of inhibiting the growth of the pathogenic microorganisms *Paenibacillus larvae* subsp. *larvae* and *Melissococcus plutonius* that, in honeybees, are the causative agent of American foulbrood and European foulbrood, respectively [49]. Hence, such protective effects can also be hypothesized for the insects studied in the present research.

ASVs of *L. rhamnosus* were also detected. Interestingly, this species of lactic acid bacteria showed a protective activity in *Galleria mellonella* larvae infected with *Staphyloccoccus aureus* or *Escherichia coli* [50].

Regarding *L. plantarum*, Agrawal and Broderick [51] recently reported its growth-promoting effect in fruit fly larvae. Additionally, Storelli et al. [52] demonstrated that, in *Drosophila*, the presence of *L. plantarum* as a commensal bacterium stimulated larval growth under conditions of nutrient scarcity.

As for *Gilliamella* spp., the novel species *G. intestine*, *G. bombicola*, *G. bombi*, and *G. mensalis* have isolated by Praet et al. [53] from wild bumblebees (*Bombus pascuorum*, *Bombus lapidaries*, and *Bombus terrestris*) gut, thus confirming the adaptation of this genus to the gut of insects. Notably, Zheng et al. [54] highlighted the role of *G. apicola* in supporting the health of its bee hosts by facilitating carbohydrate utilization.

*Weissella* species have previously been identified in various edible insect species, including *Locusta migratoria migratorioides*, *Ruspolia differens*, *Liometopum apiculatum* (both larvae and adults), and *Tenebrio molitor* larvae [4]. The presence of this genus of lactic acid bacteria in the samples analyzed in this study further reinforces *Weissella*’s status as one of the most well-adapted microorganisms to the insect environment.

Regarding Acetobacteraceae, *Acetobacter pomorum* has been shown to play a significant role in complementing fly auxotrophies by synthesizing several amino acids [55]. Additionally, *Acetobacter* species have been reported as symbionts of the Camellia weevil, *Curculio chinensis*, where they were capable of degrading tea saponins produced by the host trees [55].

Although *Streptococcus* species have already been detected in edible insects [4], to the authors’ knowledge *S. thermophilus* has never been detected in insects or edible insects before. Hence, the origin of this lactic acid bacteria species in the samples herein studied deserves to be clarified in further studies.

The Enterobacteriaceae ASVs identified in this study likely originated from the gut of the analyzed insect species or from cross-contamination during handling and sale. However, viable Enterobacteriaceae cells were detected only in black scorpion samples. For the other insect species analyzed, it is likely that the results were due to the amplification of DNA from dead microbial cells.

*Alcaligenes* was detected as minority species in samples of rhino beetle adults and in the two types of silkworm pupae (both from Bangkok and Koh Samui). On note, this bacterial genus has been reported as commonly associated to the gut of Coleoptera and Lepidoptera (e.g., muga silkworm) [56,57]. The same analyzed insects also exhibited the presence of *B. thermosphacta*, *Psychrobacter* spp, and *S. saprophyticus*. These spoilage bacteria were likely introduced through environmental contamination at the cooking site or market stalls.

Regarding the presence of *L. melliventris*, exclusively detected in samples of giant waterbugs and black scorpions, this species of lactic acid bacteria has already been detected in *A. mellifera* [37]. Therefore, it is likely that, together with *A. mellifera*, other insect species can be natural niches for *L. melliventris*.

*Pediococcus* was detected in samples of black scorpions. Of note, the presence of pediococci has already been reported in the edible insects including *Locusta migratoria*, *Acheta domesticus*, *Gryllus campestris*, and *Tenebrio molitor* [4].

## 5. Conclusions

The results obtained in the present study shed a first light on the microbiota occurring in ready-to-eat edible insects sold by street vendors in Thailand, thus representing an advancement in understanding microbial risks associated with this peculiar food matrix.

As a positive remark, no safety concerns were emerged from the culture-dependent analyses that showed the absence of the major foodborne pathogens *Listeria monocytogenes* and *Salmonella* spp., as well as of spore-forming bacteria.

Except for black scorpions, which were sold after boiling, hygiene indicator microorganisms such as Enterobacteriaceae were below detection limits in all the fried samples. This suggests that the frying process was more effective than boiling in reducing the microbial load.

Interestingly, metataxonomic analysis confirmed the absence of pathogenic taxa in the studied samples, with lactic acid bacteria unexpectedly making up the largest portion of the core microbiota. However, future studies are needed to provide further clarification on the origin of the microbial contamination herein disclosed. More in-depth analyses with larger sample sizes and higher resolution to distinguish bacteria at the species level will help ensure the food safety of edible insects and insect-based food products.

## Figures and Tables

**Table 1:** Water activity (a_w_) and viable counts of edible insects sold by street vendors in Thailand.

Sample	a_w_	Enterobacteriaceae	*Escherichia coli*	*Staphylococcus aureus*	Total Mesophilic Aerobes	Sulfite- Reducing Clostridia Viable Cells	Sulfite- Reducing Clostridia Spores	*Bacillus cereus*	*Listeria* *monocytogenes*	*Salmonella* spp.
A	0.97 ± 0.00 ^a^	<1	<1	<1	8.13 ± 0.05 ^a^	<1	<1	<1	Absent in 25 g	Absent in 25 g
B	0.94 ± 0.01 ^b^	<1	<1	<1	8.18 ± 0.02 ^a^	<1	<1	<1	Absent in 25 g	Absent in 25 g
C	0.93 ± 0.00 ^b^	<1	<1	<1	8.05 ± 0.02 ^a^	<1	<1	<1	Absent in 25 g	Absent in 25 g
D	0.92 ± 0.01 ^b^	<1	<1	<1	8.24 ± 0.01 ^a^	<1	<1	<1	Absent in 25 g	Absent in 25 g
E	0.91 ± 0.01 ^b^	4.05 ± 0.02 ^a^	<1	<1	8.02 ± 0.02 ^a^	<1	<1	<1	Absent in 25 g	Absent in 25 g

Values of viable counts are expressed as Log cfu g^−1^ ± standard deviation of two replicates. For each parameter, values with different superscript letters are significantly different (*p* < 0.05) according to the Tukey–Kramer’s (HSD) test. A—rhino beetle (*Hyboschema contractum*) adults collected in Koh Samui. B—silkworm (*Bombyx mori*) pupae collected in Bangkok. C—silkworm (*B. mori*) pupae collected in Koh Samui. D—giant waterbugs (*Belostomatidae*) adults collected in Koh Samui. E—black scorpions (*Heterometrus longimanus*) collected in Bangkok.

## Data Availability

The data supporting the findings of this study are available from the corresponding author upon reasonable request.

## Supplementary Materials

The following supporting information can be downloaded at: https://www.mdpi.com/article/10.3390/insects16020122/s1, Table S1: Relative frequency of bacterial ASVs of edible insect samples.
