# Supplementary material for: Deciphering the Microbiota of Edible Insects Sold by Street Vendors in Thailand Using Metataxonomic Analysis
Source: Insects. 2025 Jan 26;16(2):122. doi: 10.3390/insects16020122 (PMC11855710; doi:10.3390/insects16020122)
Supplement: Supplementary file 1 [file insects-16-00122-s001.zip › insects-3358582-supplementary.pdf]

Table S1. Relative frequency of bacterial ASVs of edible insect samples.

	A1	A2	B1	B2	C1	C2	D1	D2	E1	E2
Acetobacteraceae	0.00	0.01	0.01	0.01	0.03	0.02	7.20	0.00	10.07	0.00
Acinetobacter	1.32	1.44	0.39	0.47	0.50	0.42	8.82	0.00	0.00	0.00
Acinetobacter pragensis	0.66	0.64	0.09	0.12	0.11	0.07	0.00	0.00	0.00	0.00
Alcaligenes	0.99	0.89	0.65	0.50	0.55	0.46	0.00	0.00	0.00	0.00
Apilactobacillus kunkeei	0.04	0.04	0.01	0.02	0.02	0.00	2.76	3.51	3.83	6.87
Bacillus	0.00	0.00	0.00	0.00	0.01	0.00	0.06	0.00	0.00	4.58
Bombella	0.00	0.00	0.00	0.00	0.00	0.00	2.56	0.00	0.00	0.00
Bombilactobacillus	0.06	0.05	0.00	0.00	0.02	0.02	11.84	0.00	7.23	0.00
Bombilactobacillus mellis	0.00	0.00	0.02	0.03	0.00	0.00	1.29	0.00	1.99	0.00
Brochothrix thermosphacta	2.07	2.04	2.50	2.46	2.60	2.34	0.00	0.00	0.00	0.00
Burkholderiaceae	0.13	0.08	0.09	0.11	0.07	0.12	0.00	0.00	0.00	0.00
Carnobacterium divergens	0.07	0.07	0.07	0.11	0.04	0.06	0.00	0.00	0.00	0.76
Coleofasciculaceae	2.85	2.36	0.60	0.63	0.70	0.59	0.00	0.00	0.14	2.29
Commensalibacter	0.00	0.00	0.00	0.00	0.01	0.00	1.70	0.00	0.00	0.00
Corynebacterium	0.06	0.02	2.63	2.39	2.71	2.23	0.06	0.00	0.00	0.00
Deinococcus	0.00	0.01	0.00	0.00	0.00	0.00	0.88	0.00	0.00	0.00
Dellaglioa algida	19.94	21.13	39.42	39.37	37.80	39.96	0.26	8.77	0.00	7.63
Desulfobacterales	0.00	0.00	0.00	0.00	0.00	0.00	1.03	0.00	0.00	0.00
Enterobacterales	3.23	3.53	0.15	0.11	0.14	0.14	0.00	0.00	0.00	0.00
Enterobacteriaceae	0.53	0.39	0.30	0.29	0.24	0.25	5.00	17.54	8.79	0.76
Exiguobacterium acetylicum	0.19	0.22	0.16	0.23	0.20	0.15	0.00	0.00	0.00	0.00
Gilliamella	0.04	0.04	0.05	0.03	0.03	0.03	11.25	22.81	17.45	8.40
Lacticaseibacillus rhamnosus	0.00	0.00	0.00	0.01	0.01	0.01	0.00	3.51	0.00	0.76
Lactiplantibacillus plantarum	0.00	0.01	0.01	0.01	0.00	0.02	6.70	0.00	0.71	0.00
Lactobacillaceae	0.00	0.01	0.00	0.00	0.00	0.00	0.56	0.00	4.82	0.00
Lactobacillus	0.00	0.00	0.00	0.00	0.00	0.00	4.11	0.00	6.52	5.34
Lactobacillus apis	0.11	0.13	0.05	0.12	0.07	0.09	15.66	10.53	23.55	34.35
Lactobacillus helsingborgensis	0.00	0.00	0.01	0.00	0.00	0.00	0.68	0.00	1.13	3.05
Lactobacillus helveticus	0.22	0.27	0.00	0.04	0.01	0.03	0.00	0.00	0.00	0.00
Lactobacillus melliventris	0.00	0.00	0.00	0.00	0.00	0.00	1.00	8.77	2.55	0.00
Lactococcus spp.	0.61	0.66	0.03	0.07	0.04	0.05	0.00	3.51	0.00	0.00
Latilactobacillus curvatus	3.20	3.35	10.97	11.20	11.58	10.99	0.09	3.51	0.00	2.29
Latilactobacillus sakei	48.84	48.02	22.78	24.06	23.05	24.02	0.24	8.77	0.99	10.69
Leuconostoc inhae	9.04	8.92	2.38	2.37	2.59	2.47	0.00	0.00	0.14	0.00
Levilactobacillus brevis	0.00	0.00	0.00	0.00	0.00	0.00	0.00	0.00	2.70	0.00
Mammaliicoccus vitulinus	0.00	0.00	0.18	0.15	0.15	0.12	0.00	0.00	0.00	0.00
Pediococcus	0.00	0.00	0.00	0.00	0.00	0.00	2.47	0.00	0.00	0.00
Pediococcus pentosaceus	0.00	0.00	0.00	0.00	0.00	0.00	12.67	0.00	0.00	0.00
Pseudomonas	3.02	2.88	1.28	0.96	1.17	0.94	0.29	0.00	0.00	0.00
Psychrobacter	0.45	0.57	2.01	1.93	2.11	1.88	0.00	0.00	0.00	0.00
Rahnella	0.00	0.00	0.00	0.00	0.00	0.00	0.50	0.00	0.00	0.00
Raoultella planticola	0.00	0.01	0.00	0.00	0.00	0.00	0.00	0.00	0.00	6.11
Snodgrassella alvi	0.00	0.00	0.00	0.00	0.00	0.00	0.00	0.00	2.98	0.00
Staphylococcus	0.01	0.00	3.49	2.83	3.62	2.84	0.00	0.00	0.00	0.00
Staphylococcus equorum	0.00	0.00	0.84	0.81	0.82	0.80	0.03	0.00	0.00	0.00
Staphylococcus saprophyticus	0.01	0.01	2.66	2.43	2.48	2.69	0.00	0.00	0.00	0.00
Staphylococcus succinus	0.00	0.00	0.19	0.15	0.20	0.22	0.00	0.00	0.00	0.00
Streptococcus thermophilus	0.24	0.23	0.16	0.14	0.12	0.17	0.15	8.77	0.00	3.05
Weissella	0.97	1.01	5.03	5.08	5.21	5.11	0.09	0.00	0.00	0.00
Weissella koreensis	0.00	0.00	0.00	0.00	0.00	0.00	0.00	0.00	3.12	3.05
